# Developing Behavioral Markers of Social Function in Aging: A Methodology Using Pitch Mimicry

**DOI:** 10.1093/geroni/igab046.3585

**Published:** 2021-12-17

**Authors:** Christine Williams, Emmanuelle Tognoli, Alice Wead, Christopher Beetle, Joseph McKinley

**Affiliations:** 1 Florida Atlantic University, Boca Raton, Florida, United States; 2 Florida Atlantic University, BOCA RATON, Florida, United States

## Abstract

The Covid pandemic brought to the forefront the crucial role of social interactions for society at large and in gerontological practice. Social interactions play a paramount role in preserving cognitive reserve in older adults. They rely on neurobehavioral processes that are complex (engage large parts of the brain and demand integrity of multiple perceptuomotor, attentional, cognitive and memory functions). Pitch mimicry is a well-known and spontaneously arising social phenomenon that requires the integrity of numerous processes of the brain, and we hypothesize that it constitutes a potentially sensitive behavioral marker of neurodegeneration in Alzheimer’s Disease and Related Dementias (ADRD). We developed and validated a series of algorithms to parse verbal exchanges between people and quantify the level of mimicry that each exhibit with their partners. Those algorithms are based on silence thresholding, carefully parametrized CEPSTRAL algorithms for automatic pitch estimation and Synchrosqueezing Transform for validation. We introduce a theoretical model to compare our estimates of pitch mimicry with model’s expectations based on the null hypothesis that its neurobehavioral pathways retain their integrity. Our method will allow researchers to study the evolution of pitch mimicry in aging individuals and its sensitivity to diverse social contexts, including those preserving lasting social engagement. Our method will also allow us to test the hypothesis that Pitch Mimicry is a sensitive behavioral marker of dementia, a condition characterized by a breakdown in social relatedness.

